# Protective device in cardiopulmonary resuscitation with its hand positioning barrier device of international class: A61H/31/00

**DOI:** 10.1016/j.ahjo.2025.100629

**Published:** 2025-09-30

**Authors:** Hamed Tavan

**Affiliations:** a Student Research Committee, Faculty of Nursing and Midwifery, Ilam University of Medical Sciences, Ilam, Iran; b Health Technology Incubation Center, Ilam University of Medical Sciences, Ilam, Iran

Dear Editor,

Cardiopulmonary arrest (CPA) can occur suddenly in diverse environments and is a major contributor to global mortality [1]. CPA represents one of the most urgent medical emergencies, arising in both out-of-hospital settings, such as public spaces, and in-hospital scenarios [2]. Cardiopulmonary resuscitation (CPR) remains the primary intervention; however, it is often associated with complications, including rib fractures and dental injuries. The present invention aims to mitigate these adverse effects by employing a specialized protective device.

## 1 Solution to the technical problem with a detailed explanation of the invention

With the advancement of medical science and the expansion of public health infrastructure, there is a growing demand for devices that reduce physical injuries during CPR, such as rib fractures and crush injuries. The present invention introduces a CPR hand-positioning barrier device designed to enhance patient safety by minimizing injury severity and improving the efficiency of CPR, thereby providing significant benefits to both patients and healthcare providers.

## 2 Figure 1: protective device in CPR with holder

The device is composed of elements engineered to conform to the sternum—a dagger-shaped bone approximately 15–20 cm in length, located centrally in the thoracic cavity, articulating laterally with the costal cartilages and superiorly with the clavicle. The protective device extends from the lower sternum near the xiphoid process to the upper rib articulations (ribs 1–9), offering targeted coverage and support to enhance CPR performance while reducing complications.

This device offers the possibility of successful resuscitation of a cardiac arrest with fewer complications.

Fig. 1A: Longitudinal Section (Chest to Abdomen)

1. Upper area: Hand positioning area for compressions.
2. Lower area: Junction with the sternum.
3. Lateral coverage: Stabilizes the device on both sides of the chest.

**Fig. 1A:**
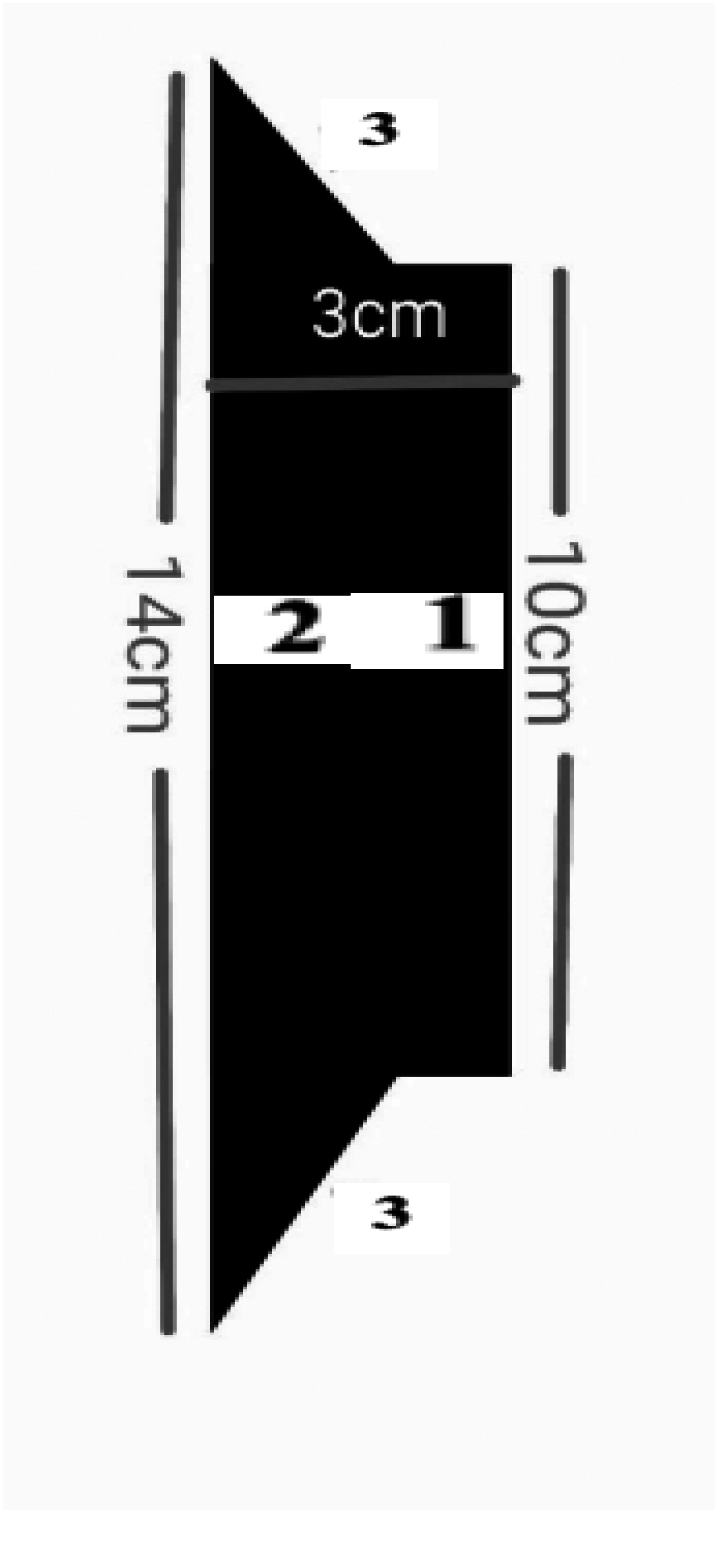
Longitudinal section (chest to abdomen).

Fig. 1B: Top View

1. Upper section: Hand positioning area.
2. Chest orientation: Maintains balance between upper/lower and left/right regions.
3. Upper linkages: Connect to lateral chest indicators.
4. Lower linkages: Fasten to lateral chest indicators.
5. Side labels: Enhance stability, precision, and balance during CPR.

**Fig. 1B:**
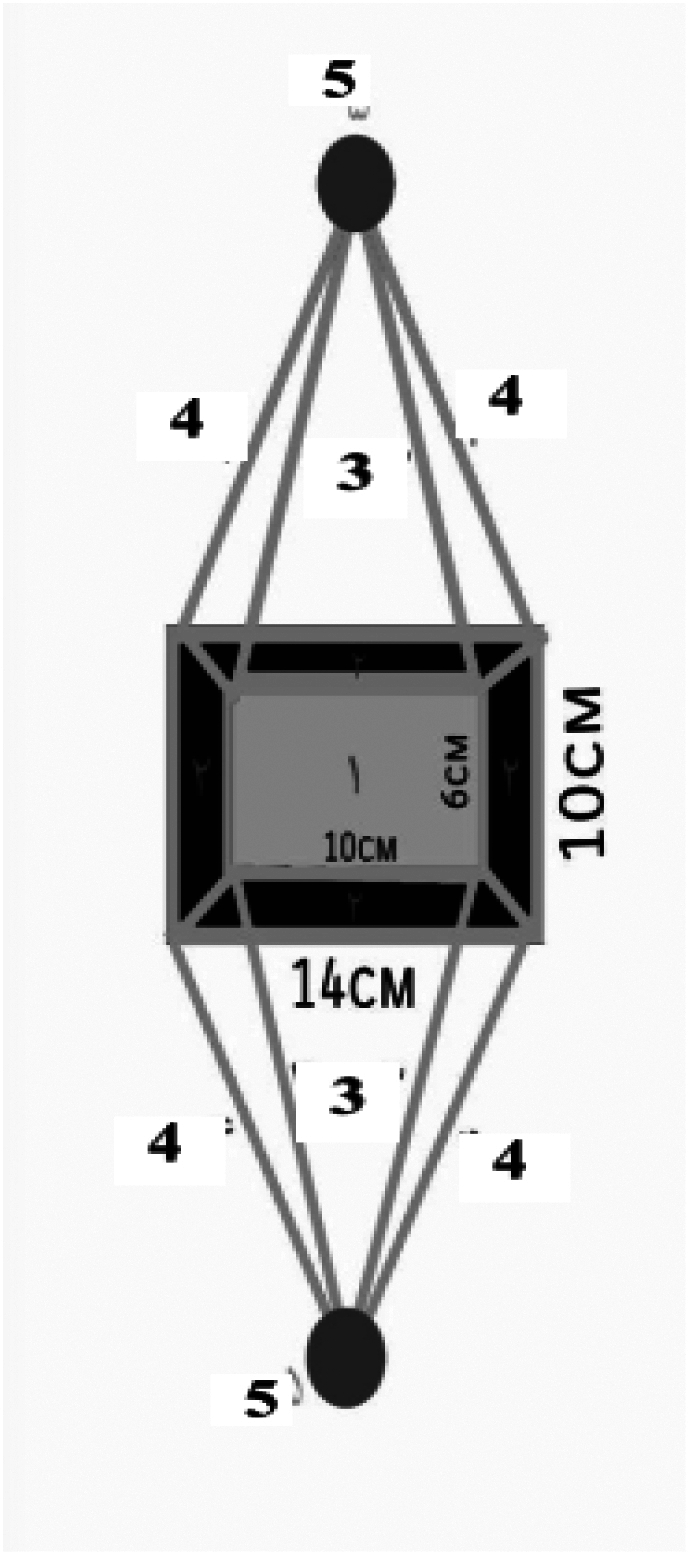
Top view.

## 3 Advantages of the invention

This device significantly reduces physical complications related to CPR, such as rib fractures and sternum injuries, while potentially improving survival rates. It is suitable for use in hospitals and public facilities, including sports arenas and educational institutions.

That this device would most likely be used in hospital and out of hospital in public facilities such as sporting grounds and schools.

Key benefits include:

1. Consistency: Fixed positioning eliminates delays during rescuer transitions.
2. Anti-slip design: Textured pad prevents hand slippage and rib damage.
3. Rib stabilization: Adhesive elements beneath the device reduce pressure on chest muscles, lowering fracture risk.
4. Force optimization: Channels compression directly to the sternum, maximizing cardiac output.
5. Pressure control: Separation of upper and lower pads prevents sudden excessive force.
6. Safety barrier: Minimizes direct contact between rescuer and patient.
7. Public training utility: Compatible with AEDs for layperson use.
8. Enhanced chest compressions: Reduces delay in resuming compressions after rescue breaths.
9. Portability: Lightweight, compact, and easily stored in emergency kits.
10. Tissue protection: Unique structure shields the sternum and ribs from injury.

Claims

1. Stabilization: Lower section secures intercostal joints; upper flexible section distributes compressive forces evenly.
2. Barrier function: Prevents direct fingertip or nail contact with the patient's chest, enhancing safety and hygiene.

## 4 Study limitations

The device has not yet undergone extensive evaluation in human subjects. Testing has primarily involved mannequins and anatomical models, demonstrating improved compression accuracy, higher compression frequency, and reduced rib fracture incidence. The lack of large-scale clinical trials constitutes a limitation, and future studies involving cadavers or clinical participants are planned where ethically and legally permissible.

## Ethical and legal considerations

Ethical issues (Including plagiarism, informed consent, misconduct, data fabrication and/or falsification, double publication and/or submission, redundancy, etc.) have been completely observed by the authors.

## Declarations ethics approval and consent to participate

This study was approved (Ethical code: A 61H/31/00) by the Intellectual property system.

## CRediT authorship contribution statement

**Hamed Tavan:** Writing – review & editing, Writing – original draft, Visualization, Validation, Supervision, Software, Resources, Project administration, Methodology, Investigation, Funding acquisition, Formal analysis, Data curation, Conceptualization.

## Consent for publication

Not applicable.

## Funding

Intellectual property system.

## Declaration of competing interest

This device is the invention of the author. The device is not commercially available at the time of submission of manuscript.

## Data Availability

The datasets used and/or analyzed during the current study are available from the corresponding author on reasonable request.
